# EEG-based emotion recognition using hybrid CNN and LSTM classification

**DOI:** 10.3389/fncom.2022.1019776

**Published:** 2022-10-07

**Authors:** Bhuvaneshwari Chakravarthi, Sin-Chun Ng, M. R. Ezilarasan, Man-Fai Leung

**Affiliations:** ^1^School of Computing and Information Science, Faculty of Science and Engineering, Anglia Ruskin University, Cambridge, United Kingdom; ^2^Department of Electronics and Communication Engineering, Vel Tech Rangarajan Dr. Sagunthala R&D Institute of Science and Technology, Chennai, India

**Keywords:** deep learning, electroencephalography, emotion recognition, neural networks, machine learning

## Abstract

Emotions are a mental state that is accompanied by a distinct physiologic rhythm, as well as physical, behavioral, and mental changes. In the latest days, physiological activity has been used to study emotional reactions. This study describes the electroencephalography (EEG) signals, the brain wave pattern, and emotion analysis all of these are interrelated and based on the consequences of human behavior and Post-Traumatic Stress Disorder (PTSD). Post-traumatic stress disorder effects for long-term illness are associated with considerable suffering, impairment, and social/emotional impairment. PTSD is connected to subcortical responses to injury memories, thoughts, and emotions and alterations in brain circuitry. Predominantly EEG signals are the way of examining the electrical potential of the human feelings cum expression for every changing phenomenon that the individual faces. When going through literature there are some lacunae while analyzing emotions. There exist some reliability issues and also masking of real emotional behavior by the victims. Keeping this research gap and hindrance faced by the previous researchers the present study aims to fulfill the requirements, the efforts can be made to overcome this problem, and the proposed automated CNN-LSTM with ResNet-152 algorithm. Compared with the existing techniques, the proposed techniques achieved a higher level of accuracy of 98% by applying the hybrid deep learning algorithm.

## Introduction

Neuroscience has played an important role in artificial intelligence (AI) history. Emotions have a significant influence on humans’ daily activities, causing psychological changes. Mental health is crucial for humans because it has an impact on their entire lives. We need a healthy mind to have a healthy life (Scherer, 2005; Verduyn et al., 2009, 2015; Hakim et al., 2013). Therefore, we must keep track of psychological changes regularly. Researchers have recently started to recognize brain impulses as a means of analyzing an individual’s mind or emotional condition. Electroencephalography (EEG) is a method of recording electrical activity in the brain via electrophysiological monitoring (Codispoti et al., 2009; Steinberg et al., 2013; Kuppens and Verduyn, 2015). Therefore, although jumper wires are sometimes used, such as in electrocorticography, placing electrodes along the scalp is unobtrusive. EEG attempts to measure voltage fluctuations in the primary ventral striatum generated by the cationic current. EEG is a medical term that refers to tracking the brain’s regular electrical activity over time using numerous electric pulses on the scalp (Russell, 1980; Ekman, 2014). Despite this, methods for high-resolution anatomical imaging, such as magnetic resonance (MRI) with computed tomography (CT), have already been developed, and their usage has increased. EEG remains an essential research and diagnostic tool despite its limited spatial resolution. It is one of the few transportable modalities available, with a temporal resolution of milliseconds that Computed Tomography (CT), Positron Emission Tomography (PET), and Magnetic Resonance Imaging (MRI) cannot match.

Evoked potentials are an EEG derivative that entails averaging EEG activity moments to deliver a stimulus of a certain type (visual, somatosensory, or auditory). EEG reactions that are moment to more complex stimuli are event-related potentials. This method is used in cognitive neuroscience, cognitivism, and psychophysiological research.

Electroencephalography analysis obtains data from the EEG using arithmetical signal analytical techniques and computer technology (Jenke et al., 2014). EEG analysis is intended to aid researchers in learning more about the brain, assist experts in interpreting and treating patients, and enhance complex brain interface technologies. Analytical techniques in EEG have traditionally been classified into four types: time-frequency domain, spectral analysis, wavelet transform, and non-linear methods. Later methods, such as neural networks, are widely used in different applications, including signal and image reconstruction (Dai et al., 2021; Che et al., 2022), classification (Lui et al., 2021; Xu et al., 2021), non-negative matrix factorization (Che et al., 2021; Chen et al., 2022), expensive optimization (Li et al., 2022), and asset allocation (Leung et al., 2021, 2022). The convolutional neural network (CNN) methodology was widely used in deep learning research on EEG analysis before the advent of transfer learning with CNN. Deeper CNN exhibits superior decoding performance with little training in attaining accuracy on datasets (Tan et al., 2021; Yu et al., 2021). Furthermore, large EEG data, such as those of the artificial neural network (ANN) insight, necessitates secure storage density computational resources for real-time processing. A virtualized classifier for the proper processing of large EEG data has been developed to address these issues. Some of the application and their artificial intelligence detection methods are, for Epilepsy detection method is Convolutional neural network (CNN). Brain depth or coma detection method is using Canonical correlation analysis. Brain tumor is based on artificial neural network are the detection methods used in previous decades. Emotions are present in our daily lives and play an important part in non-verbal communication. Emotions can also affect the brain region that controls reasoning, judgment, and thought. Strong emotions cause temporary effects on biological functions, such as increased heart rate, flushing of the face, faster breathing, and increased blood pressure. Several upsetting variables stimulate the driver’s care.

Many academics have previously used machine learning algorithms to identify post-traumatic stress disorder (PTSD), such as support machine learning (SVM), k-nearest neighbors (KNN) classifiers, random forest, AdaBoost, decision tree, and logistic regression. In the SEED-V dataset, the machine learning algorithms are incapable of producing more accurate findings. Therefore, in this study, we propose CNN-LSTM using the ResNet152 model, a new hybrid deep learning approach that ensures predicted efficiency in extracting entropy values. In Deburchgraeve et al. (2008), the researchers identified features of prenatal seizures that a mortal spectator may detect. Neonatal seizures can be divided into two categories. Considering the aforementioned individual observer features, a fully automated recognition system was formed for each category. The first algorithm examines how high-energy EEG features are related. The second algorithm detects a high level of autocorrelation and increases low-frequency activity (8 Hz). Their method for separating two types of neonatal seizures achieved a higher sensitivity, higher PPV, and lesser true alarm rate than previously released algorithms. Cherian et al. (2011) explored the verification of a fully electronic neonatal epileptic detection from the clinician’s viewpoint. Fully automated neonatal seizure detector, combined with EEG and HRV data, detects HRV (+9.50% sensitivity, +9.70% SPE) and EEG (+14.30% SEN, +13.40% specificity) better than a vessel. In most situations, the decision-based fusion of HRV and EEG seems to be more accurate than the function-based fusion (94.30 vs. 88.60%). Data from biological measures that directly represent toxic effects and parasympathetic behavior indicators reinforce each other, and their integration enhances seizure identification, according to the findings in this study. According to Misulis et al. (2022), a compendium of EEG and seizure compounds called investigations, the EEG signal output power of variability indicates brain electrical activity. Owing to entropy increments, the EEG signals of subject areas with PTSD disease had higher irregularity. The anterior lobe of the brain, which is associated with emotional experiences, had a greater increase in entropy. Spiraled LSTM back-propagation neural network for automatic vehicle nap stage classification using solitary signals of EEG was stated by Michielli et al. (2019). Conventional machine learning-based emotion detection models have been demonstrated to effectively classify emotions based on EEG. The average accuracies of HALA, HVLV, recognition, dominance, and likability examined by DEAP’s integrated monitored neural classification algorithm are 97.39, 97.41, 98.21, 97.68, and 89%, respectively. The model was further tested using the SEED dataset to identify positive and negative emotions, and it achieved an average accuracy of 93.74%. They indicated that categorizing inner feelings with different emotions in EEG databases improved the model accuracy.

The automatic emotion recognition system based on the two light deep learning with CNN model ensemble was investigated in Qazi et al. (2019). The Mel-frequency cepstral coefficient (MFCC) is combined with bio-signals to offer an emotion recognition system. Four categories of emotions, including anxiety, calm, sadness, and happiness are identified based on the data collected during psychological emotion stimulation research. The classifiers are made up of Multilayer Perceptrons (MLP). Experimental results demonstrate that this technique may evaluate emotions based on the EEG data with an accuracy of up to 90%. This demonstrates the feasibility of employing the MFCC-MLP detection approach to elementary feelings from brain scalp EEG signals.

The function of hippocampal theta fluctuations in academic, behavioral, and characteristic performances was examined in Korotkova et al. (2018). Theta resonance is a brain rhythm associated with various behaviors and a lot of power (5–10 Hz). According to this review, theta frequency has been linked to a variety of cognitive functions. In Newson and Thiagarajan (2019), a resting-state review of EEG with psychiatric disorders was proposed. The features of electrical impulses connected to PTSD prognostic and clinical aspects were investigated using EEG in this neuroscience study. Ramzan and Dawn (2019) examined the learning-based categorization of electron energy emotion from EEG. This research aims to discover whether there are any patterns in electrical signals found by EEG that are associated with PTSD diagnosis and severity levels. Overestimated forebrain reaction time for misdirection of tasks (P3a intensity) and decreased forebrain response to event-related potentials (P3b amplitude) are the most compelling evidence of time-domain in EEG associated with event-related potentials. These results imply that some people with PTSD have attention deficiencies; thus, they find it difficult to concentrate on everyday tasks and commit in the face of potentially dangerous distractions.

According to Fitzgerald et al. (2018), brain measurements of reaction to emotions and control can be used to forecast PTSD symptoms in combat-exposed veterans. The challenge is creating gradients that vanish or explode. In Suhaimi et al. (2020), the authors used the MFCC and CNN algorithm; however, they were unstable and frequently inaccurate and could only tackle problems involving categorization and prediction. CNN and sparse encoder and DNN with heap database are explained in Liu et al. (2020). The most prevalent issues in this study are overfitting, exploding gradients, and class imbalance. CNN with ResNet50, SEED dataset, DREAMER, and AMIGOS are discussed in Topic and Russo (2021) with drawbacks, such as hardware dependency and network behavior. Wang and Wang (2021) implemented the same using SVM, KNN, naive Bayesian, and random forest algorithms; however, this ensemble is less interpretable, and the ensembled model’s output is difficult to forecast. The advantage of using this algorithm is that compared to the single contributor model, an ensemble can make more accurate predictions and produce better outcomes. In Pereira et al. (2018), CNN with SEED IV dataset is explained; the limitation of this study is that it is necessary to address possible concomitant symptoms and diseases. The main contributions of this work are listed as follows:

• Examines the limitations encountered in previous studies and yields better results in all aspects.
• Establishes an articulate person interface system that understands nonverbal data such as users’ emotions, aspirations, and attitudes by developing and proposing an emotion recognition system based on predictive modeling and cataloging methodologies.
• A hybrid deep learning approach (i.e., CNN-LSTM with ResNet-152 model) is developed to perform emotion classification using EEG signals linked to PTSD. The activity in the brain appears to have a particular behavior that changes from one individual to another, as well as from one emotional state to another emotional state.

The remainder of the paper is organized as follows. The methodology research framework with the process flow is explained in section “Research methodology.” Feature extraction and model development are explained in section “Research framework.” Section “Convolution neural network model” introduces the CNN model. Results and discussion are elaborated in section “Results and discussion,” and the conclusion is presented in section “Conclusion and future enhancement.”

## Research methodology

**FIGURE 1:**
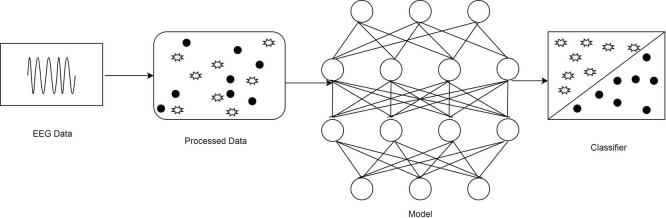
The flow of electroencephalography (EEG) data processing.

This study proposes an emotion identification system using predictive modeling and classification techniques to develop an intelligent person interaction system that helps to understand non-verbal information, such as a user’s purpose, reactions, and preferences. The flow of EEG data processing is illustrated in Figure 1. In this collected EEG data, the processing is explained. EEG Data is the collected dataset. The collected dataset is further preprocessed for normalization and filtering. The noise and artifacts can be removed using band pass filter during this preprocessing. And by using the proposed neural network model the dataset is classified accordingly.

## Research framework

**FIGURE 2:**
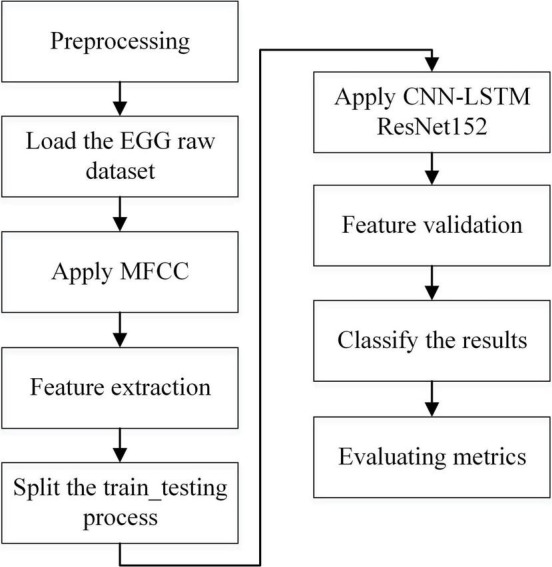
Overview of the methodology.

A composite deep learning model was created by merging a homogenous CNN and LSTM classifier with the ResNet152 model. An ensemble of many classifiers was built by adjusting the input data and changing the initial activation of the neural network’s weights. The existing SEED V EEG dataset was used in this study, which comprised emotions, such as happiness, disgust, fear, neutral, and sad. In the beginning, the preprocessing was conducted to prepare the dataset for providing input as EEG channels to the SEED-V dataset. Next, signals were extracted from the features of the MFCC (Mel Frequency Cepstral Coefficient) process using parameters of entropy, and the average power of the signal at different frequency bands for channels Frontal Pole 1 (FP1), Frontal Pole 2 (FP2), Frontal cortex (FC6), and F3 converted into the topographic map was obtained. The entropy was computed using the sample entropy method, and the Hurst exponent was computed using the R/S analysis technique. Finally, CNN and CNN-LSTM with the ResNet152 model was used to classify human emotions (e.g., fear and sadness as PTSD and happiness and neutral as Non-PSTD) and evaluate better performance metrics (e.g., the precision rate, recall rate, F1 score, and accuracy). Figure 2 explains the process flow from preprocessing to evaluation. It shows the preprocessing steps, selection of dataset, and extraction of features in the hierarchy. After the dataset has been loaded, preprocessing is the process of preparing a dataset so that it may be used in the SEED-V dataset as EEG channels such as FP1, FP2, FC6, and F3. Then we used a standard scalar with a 0–1 range of normalization. The noise and artifacts can then be filtered using the Bandpass filters spanning from 1 Hz and then 75 Hz. EEG signals for data values and data labels have been discovered.

### Feature extraction

**FIGURE 3:**
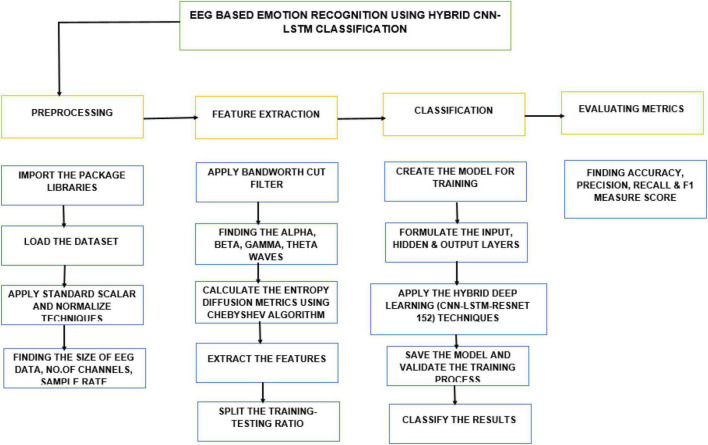
Architecture diagram for the methodology.

The overall architecture representation for the feature extraction process for the methodology and implementation is shown in Figure 3. For feature extraction preprocessing, feature extraction, classification and evaluating matrix steps to be carried out. For loading the dataset and importing the package libraries and to identify the number of channels and size of EEG data are done in preprocessing step. Feature extraction steps used to find the alpha, beta, gamma, theta waves, splitting the training testing ratios are carried out during feature extraction process. Further the dataset is given for classification and evaluating metrics which is explained in Figure 3.

### Model development and analysis

According to the proposed theory, emotion is the individual brain’s reaction to objective events. Human emotions are complicated and dynamic; thus, emotion recognition is critical for real-world applications. Recent research has shown that EEG waves may be used to determine emotions through several convolutional and machine learning techniques. However, the pattern extraction strategy used in standard machine learning techniques is often time-intensive and heavily reliant on human specialists. Using raw signal attributes and time-frequency spectra, we developed an edge deep learning algorithm as a possible solution to this problem. This study investigated the applicability of multiple algorithms in deep learning models, including LSTM, CNN, and CNN-LSTM, in the field of EEG emotional recognition. The combination-based strategy (CNN-LSTM) with the ResNet152 model was proposed after splitting the dataset into training and testing datasets in the ratio of 80:20.

## Convolution neural network model

**FIGURE 4:**
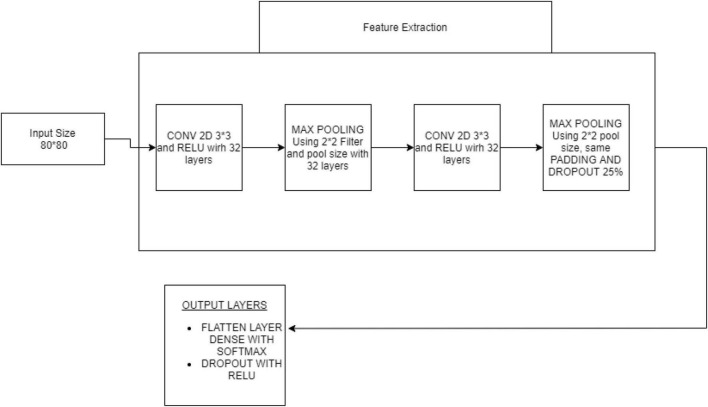
Convolutional neural network overview.

Based on a set of properties, the CNN was used to categorize objects into K separate classes. The sum of the quadratic of the closeness between an item and an appropriate cluster was employed to categorize the object. CNN is a deep learning algorithm used for evaluating picture visualization. They are referred to as shift neutral spatially ANN because of the shared-weight design of a Fourier transform, which scans the hidden units and translational affine features. Some examples of applications are image/video recognition (Guo et al., 2021), decision support, picture classification, segmentation approaches, computer vision, text analysis, central nervous system linkages, and economic time series. Multilayer perceptron’s in CNN variants have been regularized. Multilayer perceptrons are often completely connected systems, with every neuron for one layer connected to every synapse within layers above. These are “completely interrelated.” With networks, overfitting data is an issue. Regularization techniques often involve changing load as the error rate decreases and cutting connections at random. CNNs employ a different form of regularization, as shown in Figure 4. They are broken down into smaller structures imprinted in the filters to generate patterns of increasing complexity based on another person’s pattern in the data. Consequently, convents are at the extremes of connectedness and complexity. Feature extraction using CNN architecture is given in Figure 4.

In comparison to specific traditional image processing techniques, CNNs need less pre-processing. This indicates that the network evolves to improve the filtering than inversion kernels built manually in the past. This lack of reliance on prior data or human interaction during extracting features is a crucial benefit. A CNN’s inputs are a matrix of the shape (amount of photos) x (duration at high) x (photo width) x (input channels) (number of images). The images are segregated in the convolution layer, with the number of images in the shape multiplied by the feature map width and height. Kernels/Convolutional filters are characterized by their height (hyper-parameters) and width. The amount of incoming and outgoing channels is estimated. The size of the network (depth) inside the feature map must match the number of channels (depth) in the convolutional kernel/number filters. Hyper-parameters of the convolution process, such as queue length and cadence. Convolutional layers concatenate the input before parsing the output for the subsequent layer. In the experiments, the hyperparameters of the compared approaches are consistent with the best settings pointed out by the authors in their papers and codes.

Biochemical progressions prompt the fully convolutional connecting layout between neurons, which matches the anatomy of the vertebrate visual cortex. A tiny section of the visual field only reacts to feed-forward neural inputs, which constitute a small percentage of the theoretical neurons. Different neurons’ activations partially overlap, which leads to a span of the entire visual field.

Each convolution neuron can process data solely for the receptive region to which it was assigned. Additionally, two closely coupled networks may be used to recognize faces and categorize data, although they are not well suited for picture classification. When handling enormous input sizes connected with photographs, each pixel is a significant variable, necessitating the use of several hidden synapses and complex architecture. Every cell in the two tiers of ultimately linked layers has 10,000 weights for a small 100 mm by 100 mm picture. Convolution lowers the number of design variables in either case, enabling a more complex network. To tile a 55 area with the same pooling layers, for instance, regardless of picture size, only 25 input neurons are required. Applying regularized weights over fewer parameters eliminates the fading and increasing gradient issues associated with standard neural nets during training procedures.

In convolutional networks, either absolute or relative convolution may be employed to accelerate fundamental processing. By merging the responses of neuronal groups on a thin layer into a nerve cell on the subsequent layer, pooling layers minimize the amount of dataset. Local pooling connects small clusters of typical sizes 2 × 2. Global pooling affects all of the neurons in the convolution layer. Pooling is classified into two types: maximum and average. The values computed for each group with activations in the previous layer are used in max pooling, while the anticipated average is used in average pooling.

The most crucial component of a CNN is the convolutional layer. Each layer’s characteristics comprise a series of kernels or convolution layers with a small perceptron that entirely covers the complexity of the input volume. Each filter is convolved throughout the front pass over the breadth and amount of output, producing a linear model with a filtering output and input to generate a dual input vector. Consequently, the net may train an input region filter that triggers when a particular trait is detected in a defined geographic area.

The mapping for all filters along the hidden layers is concatenated to construct the entire output volume of the Fourier layer. Consequently, each piece of the generated output can be read as the outcome of a synapse processing a small subset of the stimuli and sharing data with the other synapses in the same layer.

## Results and discussion

**FIGURE 5:**
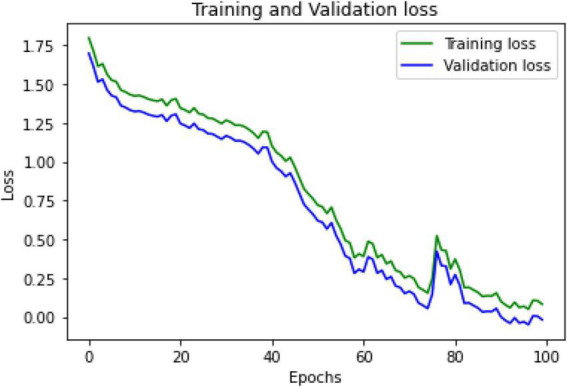
Training and validation loss.

The training and validation loss functions were calculated in the proposed model using CNN-LSTM and ResNet152 to increase the accuracy performance by training and evaluating the results. After building several layers for the model in hybrid deep learning, the loss was measured as categorical cross-entropy using the Adam optimizer. The mean squared error (MSE) and accuracy of the metrics were also measured. The epoch had a loss of 0.1 with an MSE of 0.02, as indicated in the graph shown in Figure 5.

**FIGURE 6:**
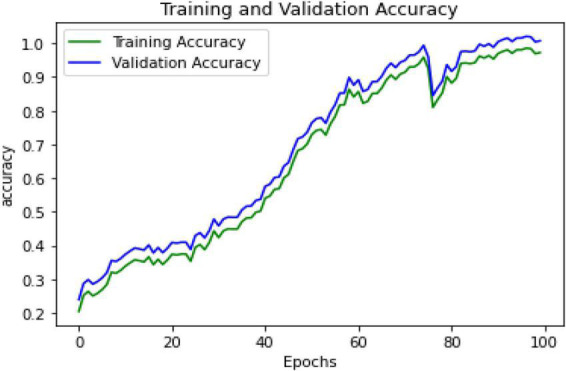
Training and validation accuracy.

The proposed hybrid deep learning strategy combining CNN-LSTM and ResNet152 model improves the accuracy to 98% in EEG-based emotion recognition connected to PTSD. Figure 6 represents the validation and training accuracies. In the training model, when the epochs reach 100, the accuracy is 0.9726 and the MSE is 0.008.

**FIGURE 7:**
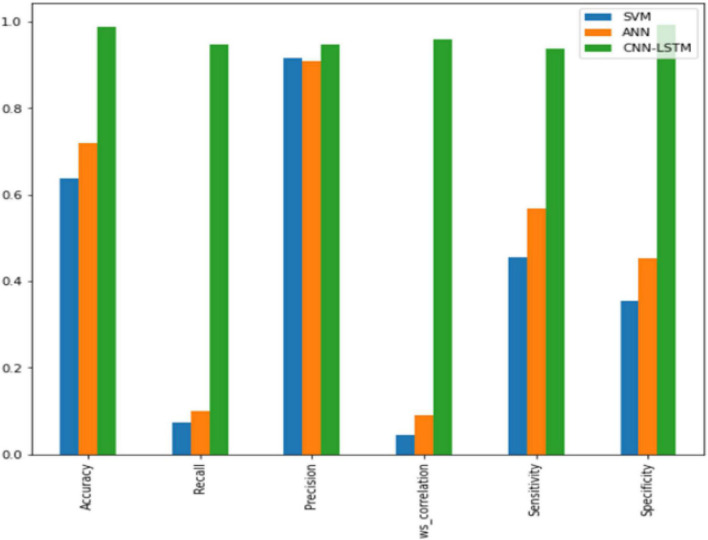
Bar chart representation of results.

After training, the model can be tested by assessing the metrics’ overall performance in comparison to algorithms, such as SVM and ANN. The proposed hybrid CNN-LSTM with the ResNet152 model performs well, with an accuracy of 98%. Figure 7 represents the overall comparison graph. The accuracy, recall, precision, and F1 score are measured to obtain the test results. The values in training can be set as array values in the range of 0–4 to predict emotions, such as happiness, sadness, fear, disgust, and neutral. Furthermore, the test can be performed accurately by improving the accuracy to 98%.

## Conclusion and future enhancement

Compared to previous methods, the proposed hybrid deep learning algorithm employing CNN-LSTM classification with the ResNet152 model has high accuracy. The comparison between the existing algorithms (i.e., SVM and ANN) and CNN-LSTM with the ResNet152 model performance is shown in Figure 6. From this figure, it is evident that the proposed hybrid deep learning algorithm outperforms the existing algorithms by improving the accuracy. The proposed technique achieved a higher level of accuracy. This study can be extended in the future to include additional behavior/emotional disorders. The study can be conducted with various samples (military personnel, IT industry people, teenagers, college students, etc.). It can also be attempted on evolving techniques on different subjects.

## Data availability statement

Publicly available datasets were analyzed in this study. This data can be found here: https://bcmi.sjtu.edu.cn/home/seed/seed-v.html.

## Author contributions

BC and ME carried out the experiment. BC wrote the manuscript with help from S-CN. S-CN and M-FL assisted in project supervision and way of writing. All authors contributed to the article and approved the submitted version.
